# 2-Oxo-2*H*-chromen-4-yl propionate

**DOI:** 10.1107/S1600536813016358

**Published:** 2013-06-19

**Authors:** Yvon Bibila Mayaya Bisseyou, Akoun Abou, Abdoulaye Djandé, Grégoire Danger, Rita Kakou-Yao

**Affiliations:** a Laboratoire de Cristallographie et Physique Moléculaire, UFR SSMT, Université Félix Houphouët Boigny, 22 BP 582 Abidjan 22, Côte d’Ivoire; b Laboratoire de Chimie Bio-organique et Phytochimie, Université de Ouagadougou 03 BP 7021 Ouagadougou 03, Burkina Faso; c Laboratoire de Physique des Interactions Ioniques et Moléculaire, UMR–CNRS 7345, Equipe "Spectrometrie et Dynamique Moléculaire", Centre Saint Jérôme, Université Aix-Marseille, Case 542 Avenue Escadrille Normandie Niemen F-13397, Marseille Cedex 20, France

## Abstract

In the title compound, C_12_H_10_O_4_, the atoms of the 2-oxo-2*H*-chromene ring system and the non-H atoms of the 4-substituent all lie on a crystallographic mirror plane. The mol­ecular structure exhibits an intra­molecular C—H⋯O hydrogen bond, which generates an *S*(6) ring. In the crystal, mol­ecules form *R*_3_^2^(12) trimeric units *via* C—H⋯O inter­actions which propagate into layers parallel to the *ac* plane. These layers are linked by weak C—H⋯O inter­actions along the [010] direction, generating a three-dimensional network.

## Related literature

For the biological activity of coumarin derivatives, see: Abernethy (1969 ▶); Wang *et al.* (2001 ▶); Yu *et al.* (2003 ▶, 2007 ▶); Vukovic *et al.* (2010 ▶). For industrial applications, see: O’Kennedy & Thornes (1997 ▶); Lakshmi *et al.* (1995 ▶). For a related structure, see: Abou *et al.* (2012 ▶). For hydrogen-bond motifs, see: Bernstein *et al.* (1995 ▶). For thermal motion of carbonyl group oxygen atoms, see: Braga & Koetzle (1988 ▶).
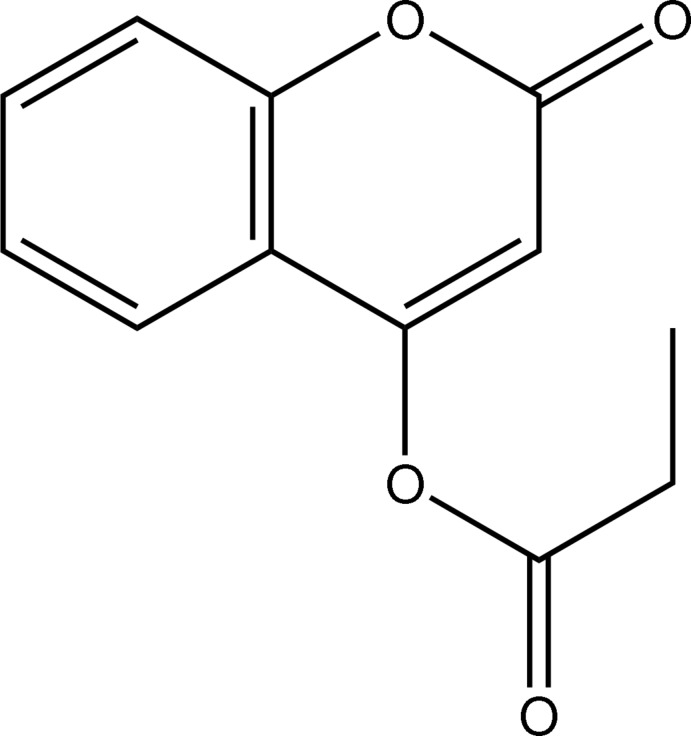


## Experimental

### 

#### Crystal data

C_12_H_10_O_4_
*M* *_r_* = 218.20
Orthorhombic, 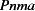

*a* = 9.2834 (3) Å
*b* = 6.7081 (2) Å
*c* = 16.8068 (6) Å
*V* = 1046.63 (6) Å^3^
*Z* = 4
Mo *K*α radiation
μ = 0.11 mm^−1^
*T* = 298 K
0.40 × 0.40 × 0.20 mm

#### Data collection

Nonius KappaCCD diffractometer
8092 measured reflections
1431 independent reflections
1155 reflections with *I* > 2σ(*I*)
*R*_int_ = 0.037

#### Refinement

*R*[*F* ^2^ > 2σ(*F* ^2^)] = 0.056
*wR*(*F* ^2^) = 0.146
*S* = 1.06
1431 reflections
98 parameters
H-atom parameters constrained
Δρ_max_ = 0.24 e Å^−3^
Δρ_min_ = −0.16 e Å^−3^

### 

Data collection: *COLLECT* (Hooft, 1998 ▶); cell refinement: *DENZO*/*SCALEPACK* (Otwinowski & Minor, 1997 ▶); data reduction: *DENZO*/*SCALEPACK*; program(s) used to solve structure: *SIR2004* (Burla *et al.*, 2005 ▶); program(s) used to refine structure: *SHELXL97* (Sheldrick, 2008 ▶); molecular graphics: *PLATON* (Spek, 2009 ▶); software used to prepare material for publication: *publCIF* (Westrip, 2010 ▶) and *WinGX* (Farrugia, 2012 ▶).

## Supplementary Material

Crystal structure: contains datablock(s) I, global. DOI: 10.1107/S1600536813016358/zq2203sup1.cif

Structure factors: contains datablock(s) I. DOI: 10.1107/S1600536813016358/zq2203Isup2.hkl

Supplementary material file. DOI: 10.1107/S1600536813016358/zq2203Isup3.cml

Additional supplementary materials:  crystallographic information; 3D view; checkCIF report

## Figures and Tables

**Table 1:** Hydrogen-bond geometry (Å, °)

*D*—H⋯*A*	*D*—H	H⋯*A*	*D*⋯*A*	*D*—H⋯*A*
C2—H2⋯O4	0.93	2.21	2.800 (3)	121
C6—H6⋯O2^i^	0.93	2.48	3.376 (3)	161
C8—H8⋯O2^ii^	0.93	2.71	3.394 (3)	131

Symmetry codes: (i) 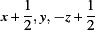; (ii) 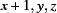.

**Fig. 1.**
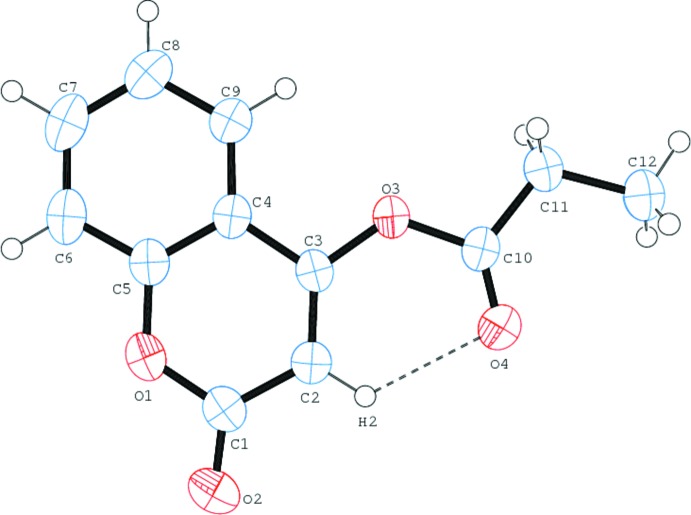
The molecular structure of the title compound and the atomic numbering scheme. Displacement ellipsoids are drawn at the 50% probability level. H atoms are shown as spheres of arbitrary radius. Dashed lines indicate a hydrogen bond.

**Fig. 2.**
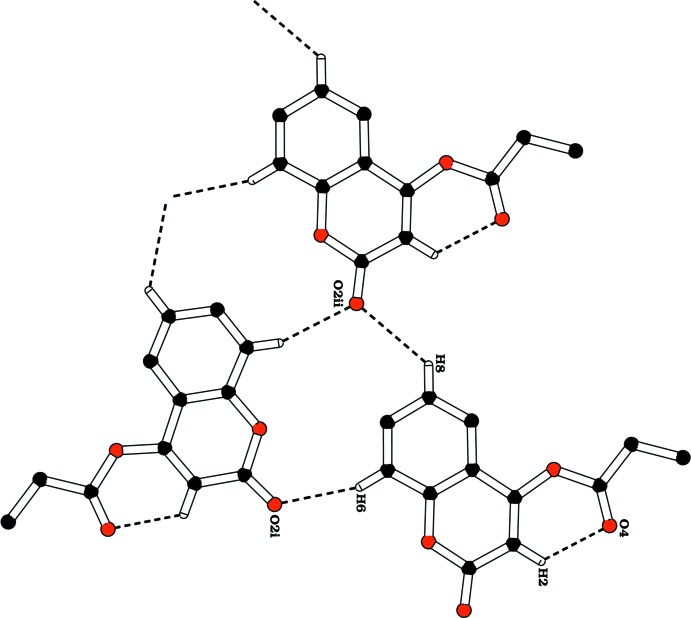
Part ot the crystal packing of the title compound showing the formation of R32(12) cyclic trimers. Dashed lines indicate hydrogen bond contacts. H atoms not involved in hydrogen bond interactions have been omitted for clarity.

**Fig. 3.**
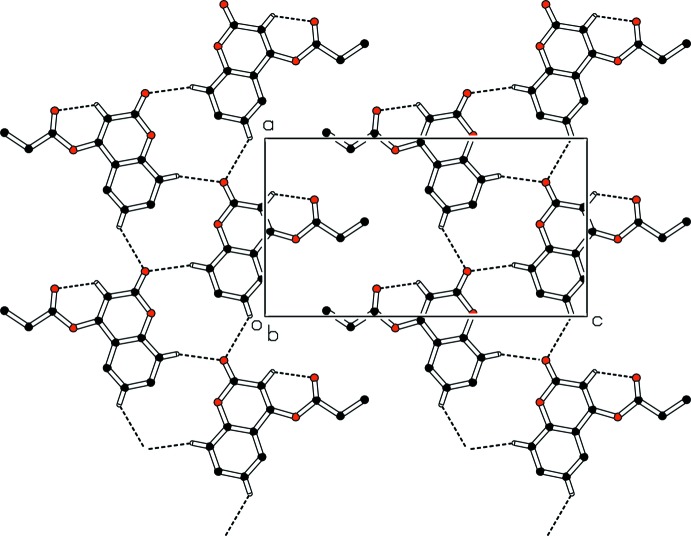
Crystal packing of the title compound viewed down the b axis, showing parallel layers in the ac plane. Dashed lines indicate hydrogen bonds. H atoms not involved in hydrogen bond interactions have been omitted for clarity.

## supplementary crystallographic information

### Comment

The 2-Oxo-2*H*-chromene ring system derivatives commonly called coumarin derivatives attracted significant attention because of their interesting biological profile including anti-HIV (Yu *et al.*, 2003; Yu *et al.*, 2007), anti-coagulant (Abernethy, 1969), anti-oxidant (Vukovic *et al.*, 2010), anti-tumor (Wang *et al.*, 2001) properties.

They found applications in cosmetic and food industries (O'Kennedy & Thornes, 1997) and are also potential laser dyes (Lakshmi *et al.*, 1995). Owing its versatile properties, coumarin ring system has become a hub nucleus in the developing of new molecules in organic, medicinal and material chemistry. We have synthesized novel coumarin derivatives substituted at position 4 in order to explore the new properties of this compound class. Herein, we report single-crystal structure of title compound.

The molecular structure of title compound (and its atomic numbering scheme) is illustrated in Fig. 1. As expected, the coumarin moiety is planar as shown in the recent X-ray diffraction analysis of 4-substituted coumarin derivative (Abou *et al.*, 2012). Analysis of bond length values of aromatic ring indicate the existence of a delocalized π-electron cloud in this one. In this structure, except the H atoms of the substituent groups, all other atoms lie in a crystallographic mirror plane (x, *y* = 1/4, z). We also note the existence of an intramolecular C—H···O hydrogen bond which generates a S(6) ring motif (Bernstein *et al.*, 1995).

In the three-dimensional crystal packing, molecules form cyclic trimers of *R*_3_^2^ (12) motifs (Bernstein *et al.*, 1995) *via* two independent intermolecular C—H···O hydrogen bond interactions along the *a* axis (Fig. 2). These trimolecular aggregates propagate into parallel layers to the *ac* plane (Fig. 3). These layers are additionally stabilized by weak intermolecular C—H···O interactions along [010] direction.

### Experimental

To a solution of propionic chloride (125 mmol) in dried diethyl ether (300 ml) was added dried pyridine (4 ml) and 4-hydroxycoumarin (120 mmol) in small portions over 30 min. The mixture was then refluxed for 3 h and poured in 300 ml of chloroform. The solution was acidified with dilute hydrochloric acid until the pH was 2–3. The organic layer was extracted, washed with water, dried over MgSO_4_ and the solvent removed. The crude product was recrystallized from acetone. Colourless single crystals of the title compound were obtained in a good yield: 69.7%; m.p. 358–359 K.

### Refinement

The structure solution program *SIR2004* (Burla *et al.*, 2005) was used to solve the structure. The .res file shows that all non H atoms lie on special positions (*x*, 1/4, *z*) with a site-occupancy factor of 0.5. As the program *SHEIXL97* (Sheldrick, 2008) will automatically work out and apply the appropriate positional, s.o.f. and *U^ij^* constraints for any special positions, we have not refined these non H atoms parameters with further constraints. However, omitted from the refinement because of bad disagreements were (7 0 4), (7 2 4), (4 1 1), (1 0 1), (0 0 2).

H atoms were placed in calculated positions [C—H = 0.93 (aromatic), 0.96 (methyl group) or 0.97 Å (methylene group)] and refined using a riding model approximation with *U*_iso_(H) constrained to 1.2 (aromatic and methylene group) or 1.5 (methyl group) times *U*_eq_ of the respective parent atom.

### Crystal data

**Table:**

C_12_H_10_O_4_	*D*_x_ = 1.385 Mg m^−^^3^
*M**_r_* = 218.20	Melting point = 358–359 K
Orthorhombic, *P**n**m**a*	Mo *K*α radiation, λ = 0.71073 Å
Hall symbol: -P 2ac 2n	Cell parameters from 8092 reflections
*a* = 9.2834 (3) Å	θ = 3.3–29.0°
*b* = 6.7081 (2) Å	µ = 0.11 mm^−^^1^
*c* = 16.8068 (6) Å	*T* = 298 K
*V* = 1046.63 (6) Å^3^	Parallelepiped, colourless
*Z* = 4	0.40 × 0.40 × 0.20 mm
*F*(000) = 456	

### Data collection

**Table:**

Nonius KappaCCD diffractometer	1155 reflections with *I* > 2σ(*I*)
Radiation source: fine-focus sealed tube	*R*_int_ = 0.037
Graphite monochromator	θ_max_ = 29.0°, θ_min_ = 3.3°
φ and ω scans	*h* = −12→12
8092 measured reflections	*k* = −8→8
1431 independent reflections	*l* = −22→22

### Refinement

**Table:**

Refinement on *F*^2^	Primary atom site location: structure-invariant direct methods
Least-squares matrix: full	Secondary atom site location: difference Fourier map
*R*[*F*^2^ > 2σ(*F*^2^)] = 0.056	Hydrogen site location: inferred from neighbouring sites
*wR*(*F*^2^) = 0.146	H-atom parameters constrained
*S* = 1.06	*w* = 1/[σ^2^(*F*_o_^2^) + (0.0589*P*)^2^ + 0.3235*P*] where *P* = (*F*_o_^2^ + 2*F*_c_^2^)/3
1431 reflections	(Δ/σ)_max_ < 0.001
98 parameters	Δρ_max_ = 0.24 e Å^−^^3^
0 restraints	Δρ_min_ = −0.16 e Å^−^^3^
88 constraints	

### Special details

**Table:**

Geometry. All s.u.'s (except the s.u. in the dihedral angle between two l.s. planes) are estimated using the full covariance matrix. The cell s.u.'s are taken into account individually in the estimation of s.u.'s in distances, angles and torsion angles; correlations between s.u.'s in cell parameters are only used when they are defined by crystal symmetry. An approximate (isotropic) treatment of cell s.u.'s is used for estimating s.u.'s involving l.s. planes.
Refinement. Refinement of *F*^2^ against ALL reflections. The weighted *R*-factor *wR* and goodness of fit *S* are based on *F*^2^, conventional *R*-factors *R* are based on *F*, with *F* set to zero for negative *F*^2^. The threshold expression of *F*^2^ > 2σ(*F*^2^) is used only for calculating *R*-factors(gt) *etc*. and is not relevant to the choice of reflections for refinement. *R*-factors based on *F*^2^ are statistically about twice as large as those based on *F*, and *R*- factors based on ALL data will be even larger.The *DENZO* image processing package used during process data may have problems with certain strong reflections. These reflections are often excluded from the data set to result in _diffrn_measured_fraction_theta_full Low (0.974 in our nvestigation study). However, it presents no problem in the refinement since the data-to-parameter ratio is superior to 10.In the initial refinement, extinction correction (EXTI) has been applied because *SHELXL* has suggested it; but in the last cycles of the refinement, the EXTI instruction has been removed because of *PLATON checkCIF* reports mentioning extinction parameter within range (2.20 σ).The non H atoms lie in the miror plane at *y* = 1/4. Therefore the *U^ij^* constraints (U_12_ = U_23_ = 0) generated automatically by *SHELXL* for this special positions (*x*, 1/4, *z*) in the space group *Pnma* is responsible for the elongated thermal ellipsoids in the [010] direction causing a large U_3_/U_1_ ratio for the average U(i,j) tensor (2.4).The low *U*_eq_ as compared to neighbors for atom C10 may be caused by the carbonyl bond in which the oxygen atom vibrates more than the carbon atom (Braga & Koetzle, 1988). Moreover, the decrease of *U*_eq_ from C12 to C10 of the propanoate substituent originates from the minor unresolved disordered H atoms bonded to the non disordered carbon atom C12 (split H atoms) revealed by manual inspection of *PLATON* (Spek, 2009) may partly justify this low *U_eq_* of C10.

### Fractional atomic coordinates and isotropic or equivalent isotropic displacement parameters (Å^2^)

**Table:**

	*x*	*y*	*z*	*U*_iso_*/*U*_eq_	Occ. (<1)
O3	0.06438 (15)	0.2500	0.59531 (8)	0.0595 (5)	
O1	−0.01804 (18)	0.2500	0.35401 (8)	0.0607 (5)	
C4	0.1469 (2)	0.2500	0.46462 (11)	0.0434 (4)	
C3	0.0233 (2)	0.2500	0.51696 (10)	0.0428 (4)	
C2	−0.1113 (2)	0.2500	0.48839 (12)	0.0481 (5)	
H2	−0.1890	0.2500	0.5233	0.058*	
C10	−0.0290 (2)	0.2500	0.65979 (11)	0.0460 (5)	
C5	0.1204 (2)	0.2500	0.38352 (12)	0.0461 (5)	
O4	−0.15508 (17)	0.2500	0.65323 (9)	0.0695 (5)	
C11	0.0580 (2)	0.2500	0.73405 (11)	0.0616 (7)	
H11A	0.1197	0.3668	0.7343	0.074*	0.50
H11B	0.1197	0.1332	0.7343	0.074*	0.50
C9	0.2892 (2)	0.2500	0.49029 (13)	0.0633 (7)	
H9	0.3096	0.2500	0.5445	0.076*	
O2	−0.25262 (19)	0.2500	0.37225 (11)	0.0921 (8)	
C1	−0.1362 (2)	0.2500	0.40383 (13)	0.0579 (6)	
C8	0.4004 (3)	0.2500	0.43576 (16)	0.0765 (9)	
H8	0.4954	0.2500	0.4532	0.092*	
C12	−0.0325 (3)	0.2500	0.80905 (13)	0.0723 (8)	
H12A	−0.0996	0.1412	0.8072	0.108*	0.50
H12B	−0.0842	0.3735	0.8129	0.108*	0.50
H12C	0.0290	0.2352	0.8546	0.108*	0.50
C6	0.2310 (3)	0.2500	0.32839 (13)	0.0603 (6)	
H6	0.2111	0.2500	0.2742	0.072*	
C7	0.3703 (3)	0.2500	0.35516 (15)	0.0687 (7)	
H7	0.4457	0.2500	0.3187	0.082*	

### Atomic displacement parameters (Å^2^)

**Table:**

	*U*^11^	*U*^22^	*U*^33^	*U*^12^	*U*^13^	*U*^23^
O3	0.0399 (8)	0.1082 (14)	0.0304 (7)	0.000	0.0025 (5)	0.000
O1	0.0549 (9)	0.0936 (12)	0.0336 (7)	0.000	−0.0011 (6)	0.000
C4	0.0423 (10)	0.0528 (11)	0.0351 (9)	0.000	0.0050 (7)	0.000
C3	0.0420 (10)	0.0554 (11)	0.0311 (8)	0.000	0.0007 (7)	0.000
C2	0.0401 (10)	0.0665 (13)	0.0376 (9)	0.000	0.0013 (7)	0.000
C10	0.0409 (10)	0.0616 (12)	0.0354 (9)	0.000	0.0062 (7)	0.000
C5	0.0485 (11)	0.0532 (11)	0.0368 (9)	0.000	0.0036 (8)	0.000
O4	0.0419 (8)	0.1227 (16)	0.0439 (8)	0.000	0.0053 (6)	0.000
C11	0.0468 (12)	0.1050 (19)	0.0331 (10)	0.000	0.0013 (8)	0.000
C9	0.0432 (11)	0.104 (2)	0.0432 (10)	0.000	0.0039 (9)	0.000
O2	0.0540 (10)	0.173 (2)	0.0496 (9)	0.000	−0.0147 (8)	0.000
C1	0.0493 (12)	0.0841 (16)	0.0402 (10)	0.000	−0.0031 (9)	0.000
C8	0.0440 (12)	0.126 (3)	0.0589 (15)	0.000	0.0111 (10)	0.000
C12	0.0577 (14)	0.123 (2)	0.0363 (10)	0.000	0.0053 (9)	0.000
C6	0.0654 (14)	0.0757 (16)	0.0397 (10)	0.000	0.0133 (10)	0.000
C7	0.0603 (14)	0.0908 (18)	0.0550 (13)	0.000	0.0232 (11)	0.000

### Geometric parameters (Å, º)

**Table:**

O3—C3	1.371 (2)	C11—H11A	0.9700
O3—C10	1.388 (2)	C11—H11B	0.9700
O1—C5	1.378 (3)	C9—C8	1.380 (3)
O1—C1	1.380 (3)	C9—H9	0.9300
C4—C5	1.385 (3)	O2—C1	1.204 (3)
C4—C9	1.390 (3)	C8—C7	1.383 (4)
C4—C3	1.446 (3)	C8—H8	0.9300
C3—C2	1.339 (3)	C12—H12A	0.9600
C2—C1	1.440 (3)	C12—H12B	0.9600
C2—H2	0.9300	C12—H12C	0.9600
C10—O4	1.176 (3)	C6—C7	1.370 (4)
C10—C11	1.487 (3)	C6—H6	0.9300
C5—C6	1.383 (3)	C7—H7	0.9300
C11—C12	1.515 (3)		
			
C3—O3—C10	125.20 (15)	C12—C11—H11A	108.9
C5—O1—C1	121.52 (16)	C10—C11—H11B	108.9
C5—C4—C9	118.32 (18)	C12—C11—H11B	108.9
C5—C4—C3	117.24 (17)	H11A—C11—H11B	107.7
C9—C4—C3	124.44 (18)	C8—C9—C4	120.3 (2)
C2—C3—O3	127.17 (17)	C8—C9—H9	119.9
C2—C3—C4	121.50 (17)	C4—C9—H9	119.9
O3—C3—C4	111.33 (16)	O2—C1—O1	116.48 (19)
C3—C2—C1	120.26 (18)	O2—C1—C2	125.4 (2)
C3—C2—H2	119.9	O1—C1—C2	118.13 (18)
C1—C2—H2	119.9	C9—C8—C7	120.0 (2)
O4—C10—O3	123.27 (18)	C9—C8—H8	120.0
O4—C10—C11	128.30 (18)	C7—C8—H8	120.0
O3—C10—C11	108.43 (16)	C7—C6—C5	118.8 (2)
O1—C5—C6	116.82 (19)	C7—C6—H6	120.6
O1—C5—C4	121.34 (18)	C5—C6—H6	120.6
C6—C5—C4	121.8 (2)	C6—C7—C8	120.8 (2)
C10—C11—C12	113.39 (19)	C6—C7—H7	119.6
C10—C11—H11A	108.9	C8—C7—H7	119.6
			
C10—O3—C3—C2	0.0	C3—C4—C5—C6	180.0
C10—O3—C3—C4	180.0	O4—C10—C11—C12	0.0
C5—C4—C3—C2	0.0	O3—C10—C11—C12	180.0
C9—C4—C3—C2	180.0	C5—C4—C9—C8	0.0
C5—C4—C3—O3	180.0	C3—C4—C9—C8	180.0
C9—C4—C3—O3	0.0	C5—O1—C1—O2	180.0
O3—C3—C2—C1	180.0	C5—O1—C1—C2	0.0
C4—C3—C2—C1	0.0	C3—C2—C1—O2	180.0
C3—O3—C10—O4	0.0	C3—C2—C1—O1	0.0
C3—O3—C10—C11	180.0	C4—C9—C8—C7	0.0
C1—O1—C5—C6	180.0	O1—C5—C6—C7	180.0
C1—O1—C5—C4	0.0	C4—C5—C6—C7	0.0
C9—C4—C5—O1	180.0	C5—C6—C7—C8	0.0
C3—C4—C5—O1	0.0	C9—C8—C7—C6	0.0
C9—C4—C5—C6	0.0		

### Hydrogen-bond geometry (Å, º)

**Table:**

*D*—H···*A*	*D*—H	H···*A*	*D*···*A*	*D*—H···*A*
C2—H2···O4	0.93	2.21	2.800 (3)	121
C6—H6···O2^i^	0.93	2.48	3.376 (3)	161
C8—H8···O2^ii^	0.93	2.71	3.394 (3)	131

Symmetry codes: (i) *x*+1/2, *y*, −*z*+1/2; (ii) *x*+1, *y*, *z*.

## References

[bb1] Abernethy, J. L. (1969). *J. Chem. Educ.* **46**, 561–568.

[bb2] Abou, A., Djandé, A., Danger, G., Saba, A. & Kakou-Yao, R. (2012). *Acta Cryst.* E**68**, o3438–o3439. 10.1107/S1600536812047666 PMC3589019 23476255

[bb3] Bernstein, J., Davis, R. E., Shimoni, L. & Chang, N.-L. (1995). *Angew. Chem. Int. Ed. Engl.* **34**, 1555–1573.

[bb4] Braga, D. & Koetzle, T. F. (1988). *Acta Cryst.* B**44**, 151–156.

[bb5] Burla, M. C., Caliandro, R., Camalli, M., Carrozzini, B., Cascarano, G. L., De Caro, L., Giacovazzo, C., Polidori, G. & Spagna, R. (2005). *J. Appl. Cryst.* **38**, 381–388.

[bb6] Farrugia, L. J. (2012). *J. Appl. Cryst.* **45**, 849–854.

[bb7] Hooft, R. (1998). *COLLECT* Nonius BV, Delft, The Netherlands.

[bb8] Lakshmi, G. S. P. B., Murthy, Y. L. N., Anjaneyulu, A. S. R. & Santhamma, C. (1995). *Dyes and Pigments* **29**, pp. 211–225.

[bb9] O’Kennedy, R. & Thornes, R. D. (1997). *Coumarins: Biology, Applications and Mode of Action* Wiley & Sons, Chichester.

[bb10] Otwinowski, Z. & Minor, W. (1997). *Methods in Enzymology*, Vol. 276, *Macromolecular Crystallography*, Part A, edited by C. W. Carter Jr & R. M. Sweet, pp. 307–326. New York: Academic Press.

[bb11] Sheldrick, G. M. (2008). *Acta Cryst.* A**64**, 112–122. 10.1107/S0108767307043930 18156677

[bb12] Spek, A. L. (2009). *Acta Cryst.* D**65**, 148–155. 10.1107/S090744490804362X PMC2631630 19171970

[bb13] Vukovic, N., Sukdolak, S., Solujic, S. & Niciforovic, N. (2010). *Arch. Pharm. Res.* **33**, 5–15. 10.1007/s12272-010-2220-z 20191339

[bb14] Wang, M., Wang, L., Li, Y. & Li, Q. (2001). *Transition Met. Chem.* **26**, 307–310.

[bb15] Westrip, S. P. (2010). *J. Appl. Cryst.* **43**, 920–925.

[bb16] Yu, D., Morris-Natschke, S. L. & Lee, K.-H. (2007). *Med. Res. Rev.* **27**, 108–132. 10.1002/med.20075 16888749

[bb17] Yu, D., Suzuki, M., Xie, L., Morris-Natschke, S. L. & Lee, K.-H. (2003). *Med. Res. Rev.* **23**, 322–345. 10.1002/med.10034 12647313

